# Research Progress on the Regulatory Effect of Curcumin on Mesenchymal Stem Cells

**DOI:** 10.3390/ijms27021015

**Published:** 2026-01-20

**Authors:** Lei Sun, Die Hu, Xinyu Dong, Ruihua Wang, Wei He, Yunjian Pan, Pingjie Li, Xuekun Xing

**Affiliations:** 1 School of Public Health, Guilin Medical University, Guilin 541199, China; 25207061018@stu.glmu.edu.cn (L.S.); 25207071007@stu.glmu.edu.cn (D.H.); 18249835129@163.com (X.D.); wruihua@outlook.com (R.W.); 18993784898@163.com (W.H.); 24207071019@stu.glmu.edu.cn (Y.P.);; 2 Guangxi Key Laboratory of Environmental Exposomics and Entire Lifecycle Heath, School of Public Health, Guilin Medical University, Guilin 541199, China

**Keywords:** curcumin, mesenchymal stem cells, proliferation, apoptosis, differentiation

## Abstract

Curcumin is the main active ingredient in Curcuma longa turmeric, with a wide range of biological effects. It shows significant therapeutic potential in the field of stem cell therapy. This article aims to explore the modulatory effects and underlying mechanisms of curcumin on mesenchymal stem cells (MSCs), providing a theoretical basis based on experimental evidence for its clinical application in regenerative medicine. First, the physicochemical properties, main pharmacological activities, and metabolic pathways of curcumin are described. Subsequently, the key molecular mechanisms by which curcumin regulates MSCs are analyzed in depth, demonstrating that curcumin can significantly promote MSC proliferation and inhibit apoptosis by modulating signaling pathways and gene expression. Additionally, curcumin directs the differentiation of MSCs into osteoblasts and chondrocytes. It also inhibits their differentiation into adipocytes, thereby regulating the physiological functions of MSCs such as proliferation, differentiation, and apoptosis. Finally, several main challenges in current research are highlighted. These include the low oral bioavailability of curcumin; the regulatory effects that vary depending on doses and microenvironmental conditions; the underlying mechanisms not being fully elucidated; the research being mostly limited to in vitro cell models and animal experiments; and the lack of quality standards and production process control systems for curcumin preparations.

## 1. Introduction

Mesenchymal stem cells (MSCs) are a type of stem cell characterized by self-renewal and multipotent differentiation capabilities. Their potential in tissue repair, immune regulation, and regenerative medicine offers promising avenues for preclinical research [1]. However, after in vitro culture passage or in vivo transplantation, MSCs often face issues such as slowed proliferation rate, decreased differentiation ability, and increased apoptosis rate, which severely limit their clinical application effectiveness [2]. In recent years, due to the low toxicity and multi-target characteristics of natural products and their derivatives, research on improving the function of MSCs has gradually become an important direction [3].

Curcumin is a natural polyphenolic compound extracted from the rhizome of “Curcuma longa”, a plant of the Zingiberaceae family. It possesses various biological activities, including anti-inflammatory, antioxidant, anti-tumor, and immune regulatory effects. As an active component of traditional Chinese medicine, studies have shown that curcumin can modulate the proliferation, differentiation, and migration of MSCs by regulating multiple signaling pathways [4]. This article aims to systematically explore the regulatory effects of curcumin on mesenchymal stem cells, analyze the limitations in current research, and evaluate future research directions, thereby providing theoretical support for the application of curcumin in stem cell therapy.

## 2. Data Sources and Retrieval Strategy

**Figure 1:**
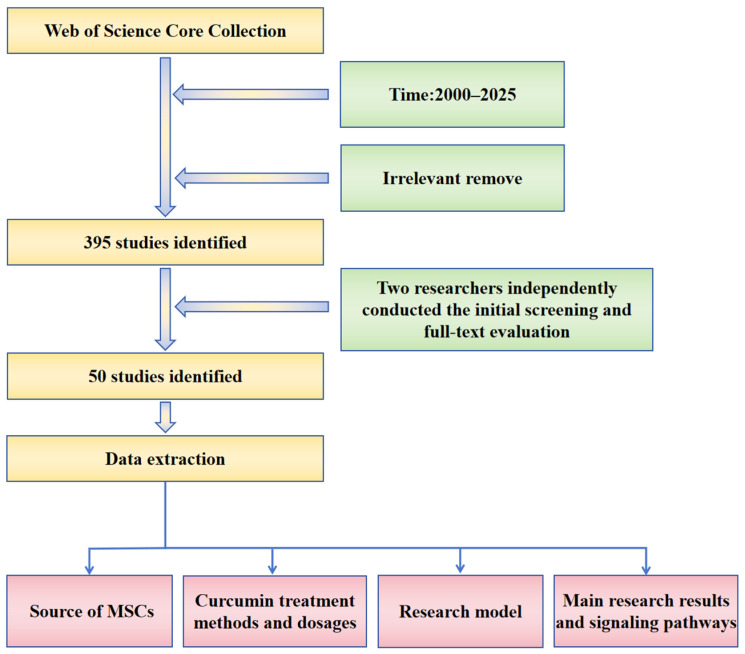
Literature retrieval flowchart.

The literature search for this review was conducted in the Web of Science core database. The time range for the search was set from 2000 to 2025. The search strategy in WOS was (ALL = (Curcumin OR Curcuma longa OR diferuloylmethane) AND ALL = (mesenchymal stem cell* OR MSC* OR mesenchymal stromal cell*)) AND ALL = (regulat* OR proliferat* OR differentiat* OR apoptos* OR migrat* OR osteogen* OR adipogen* OR chondrogen* OR immunomodulat*). Additionally, conference abstracts, case reports, and studies without full text available were excluded, resulting in a total of 395 articles. Subsequently, two researchers independently reviewed the titles and abstracts for initial screening and evaluated the full texts of potentially eligible articles. Finally, 50 articles deemed the most relevant were included in this review for in-depth analysis. The data extraction items included authors, publication year, journal, sources of MSCs, curcumin treatment methods and dosages, research models, main research results, and the signaling pathways revealed. The detailed literature retrieval strategy and the specific content of the analysis are shown in Figure 1.

## 3. Physicochemical Properties of Curcumin

Curcumin is an orange-yellow powder that is poorly soluble in water but easily soluble in organic solvents such as methanol, ethanol, and dimethyl sulfoxide (DMSO), as well as in alkaline solutions. The structure of curcumin includes unsaturated aliphatic and aromatic groups. Due to the presence of conjugated double bonds, curcumin can act as an effective electron donor to exert antioxidant and anti-inflammatory effects [5]. This structural feature also determines the specificity of its interaction with biological membranes. In plants, curcumin mainly exists as three curcuminoids, curcumin, demethoxycurcumin, and bisdemethoxycurcumin, among which curcumin accounts for 77.3% of the total content.

Modern pharmacological studies have demonstrated that curcumin exhibits multiple pharmacological effects, including anti-inflammatory, antioxidant, anticancer, anti-ulcer, antibacterial, lipid-lowering, blood glucose-lowering, bile secretion-promoting, and liver-protective activities. It has become a research hotspot in the field of natural medicine [6]. Historically, curcumin was first isolated by Vogel and Pelletier in 1815. Since then, it has been used primarily as a supplement or adjuvant in clinical treatment, rather than as a direct alternative to antibiotics [7]. Curcumin is typically administered orally and primarily absorbed in the small intestines. Its metabolites include dihydrocurcumin, tetrahydrocurcumin, hexahydrocurcumin, and octahydrocurcumin, among which tetrahydrocurcumin is the main metabolic component in the body.

## 4. The Biosafety of Curcumin

The biosafety of curcumin is a key prerequisite for assessing its potential transition from laboratory research to clinical application.

At the cellular level, curcumin exhibits low toxicity and high biocompatibility in various cell types. For example, studies have shown that curcumin does not induce significant cytotoxicity in various cell lines and maintains cell viability even at high concentrations, primarily due to its antioxidant properties, which can reduce the generation of reactive oxygen species (ROS) and oxidative stress damage [8,9]. Furthermore, curcumin can effectively alleviate sodium fluoride-induced toxicity in non-mammalian models such as Caenorhabditis elegans by exerting cytoprotective effects through regulation of the MAPK signaling pathway and maintenance of mitochondrial function [10]. However, some studies indicate that the effects of curcumin are influenced by cell type and culture conditions. For instance, in certain cancer cells, high doses of curcumin can induce apoptosis. This effect is often regarded as a component of its anticancer activity rather than a general toxicity [11,12].

In animal models, the safety of curcumin has been widely validated. Multiple acute or sub-chronic toxicity experiments conducted in rats and mice showed that oral or injected administration of curcumin (doses up to 100–200 mg/kg) did not cause significant organ damage, abnormal hematological parameters, or mortality events [11,13,14]. For example, in rat models, after intraperitoneal injection of liposomal curcumin, no pleural or pulmonary pathological changes were observed; the morphology of red blood cells was normal, indicating its good local and systemic tolerance [14]. At the same time, curcumin also exhibited protective effects, such as alleviating cisplatin- or cadmium-induced hepatotoxicity and nephrotoxicity, with mechanisms involving the inhibition of inflammatory factors (such as NF-κB) and enhancement of antioxidant enzyme activity [9,15]. These studies collectively confirm the safety of curcumin at conventional doses. Nevertheless, there are certain differences in animal experimental results. Long-term high-dose studies have indicated that when doses exceed 100 mg/kg (converted based on body surface area, which far exceeds conventional dietary supplement doses), there may be a slight increase in liver weight in experimental animals, but this is usually not accompanied by significant elevations in markers of liver function damage [16]. When the dose of curcumin exceeds 500 mg/kg, it can cause mild gastrointestinal discomfort or weight loss, which is related to species specificity, route of administration, or low bioavailability of curcumin [13,17]. These studies suggest that the toxic effects of curcumin are dose-dependent, and under long-term exposure to extremely high doses, attention should be paid to its potential burden on the liver and gastrointestinal tract. However, this high-dose scenario differs significantly from the doses used in routine clinical applications, necessitating an objective distinction between its toxicological significance and practical application value.

Clinical research further supports the safety of curcumin. Multiple clinical trials indicate that oral curcumin (doses up to 6 g/d) is safe in the short term (4–12 weeks). The main adverse reactions are limited to mild gastrointestinal symptoms, such as diarrhea or nausea, and no severe organ toxicity or hematological abnormalities have been observed [13,18]. For example, in patients with migraine, an eight-week supplementation of a phosphatidylcholine–curcumin complex showed no adverse reactions [19]. Additionally, a double-blind randomized controlled trial in patients with hand osteoarthritis found that daily oral administration of 170 mg curcumin for three months was well tolerated, except for one patient who withdrew from the study due to gastrointestinal side effects [20]. However, some studies have reported that curcumin at very high doses (above 8 g/d) may affect drug-metabolizing enzymes, such as cytochrome P450 (CYP450) enzymes, potentially leading to interactions with other medications [8,13]. Moreover, variability in clinical outcomes may arise from differences in trial designs, patient populations—including variations in liver and kidney function—and the purity of curcumin formulations.

Overall, cell and animal studies fully support the biosafety of curcumin, with mechanisms mainly involving antioxidant and anti-inflammatory effects. Although clinical safety has been preliminarily confirmed in small-scale or short-term studies, the effects of long-term use and high doses of curcumin remain uncertain. Future research should focus on further clarifying the long-term safety profile of curcumin in various populations through large-scale, high-quality randomized controlled trials. Additionally, studies should explore the drug interaction mechanisms to guide its safe and precise application in clinical practice and healthcare settings.

## 5. Delivery Systems of Curcumin

Curcumin, a natural compound with broad pharmacological activity, has its clinical applications greatly limited by poor water solubility, low oral bioavailability, and chemical instability. To overcome these obstacles, various advanced delivery systems have been developed; among them, nano-curcumin, liposomal curcumin, and exosomal curcumin represent three key types. The bioavailability and pharmacokinetics of different delivery systems vary significantly, and their safety profiles also differ accordingly.

Nanocurcumin refers to curcumin encapsulated in various nanocarriers. Common carriers include solid lipid nanoparticles (SLNs), polymer nanoparticles, and others. For example, solid lipid nanoparticles loaded with curcumin can achieve an encapsulation rate of up to 70% and continuously release the drug over 12 h [21]. In wound healing studies, novel self-assembled curcumin-loaded hyaluronic acid–glycerol nanoparticles have shown good therapeutic effects in both healthy and diabetic rat models [22]. Polybutylcyanoacrylate (PBCA) polymer nanoparticles can also effectively encapsulate curcumin and release it more rapidly in acidic environments, indicating their suitability for intracellular delivery [23]. Additionally, carrier-free nanoparticles, such as those formed by the self-assembly of curcumin and paclitaxel, represent another strategy; their formation relies on the synergistic effects of curcumin derivatives [24]. The core advantage of nanocurcumin lies in significantly improving the dispersion and dissolution rate of curcumin in aqueous media by increasing the specific surface area, thereby promoting its gastrointestinal absorption. Furthermore, nanoscale properties enable passive accumulation in pathological sites such as tumor tissue through the enhanced permeability and retention (EPR) effect. However, simple nanonization lacks active targeting ability. Moreover, nanoparticles are easily cleared by the mononuclear phagocyte system (MPS) in vivo, and their physical stability may face challenges during long-term storage, with risks of aggregation or Ostwald ripening.

Liposomal curcumin refers to the encapsulation of curcumin in vesicles formed by phospholipid bilayers. Liposomes are one of the most widely studied and well-established nanocarriers. Their main advantages lie in excellent biocompatibility and biodegradability; moreover, the high similarity of their phospholipid components to cell membranes facilitates cellular uptake and fusion. The lipid bilayer effectively isolates the encapsulated curcumin from the external environment, protecting it from degradation and prolonging its circulation time. Surface modifications, such as polyethylene glycol (PEG) to achieve an “invisible” effect that avoids uptake by the MPS, or the attachment of targeting ligands such as folate and antibodies, enable active targeted delivery. Studies have confirmed that liposomal curcumin is 2- to 6-fold more potent than free curcumin in inhibiting the production of the macrophage inflammatory factors nitric oxide (NO), interleukin-1 beta (IL-1β), and tumor necrosis factor-alpha (TNF-α) [25]. This enhanced effect is attributed to the ability of liposomes to enhance cellular uptake [26]. However, liposomes also have prominent drawbacks, including susceptibility of lipids to oxidation, potential vesicle fusion or drug leakage, and the challenge of maintaining consistency and stability between batches during large-scale production.

The exosome curcumin delivery system utilizes endogenous extracellular vesicles (30–150 nm) as carriers. Exosomes are formed inside cells within endosomal compartments and secreted via exocytosis, naturally carrying proteins, RNA, and lipids from donor cells. This characteristic endows them with unparalleled biological advantages (extremely low immunogenicity; excellent biocompatibility; and inherent targeting and homing capabilities), as their surface proteins can recognize and bind to specific receptor cells, thereby efficiently crossing biological barriers such as the blood–brain barrier. Loading curcumin into exosomes results in a highly stable formulation that can precisely deliver drugs to target cells; this greatly improves therapeutic efficiency and reduces side effects. The advantages of this system lie in its potential for higher biocompatibility, low immunogenicity, and natural targeting. For example, using engineered cell-derived nano-sized vesicles—such as vesicles derived from gene-engineered cells overexpressing PD-1—to encapsulate curcumin-loaded PLGA nanoparticles has shown synergistic effects in immunotherapy [27], indicating the tremendous potential of exosome-based delivery systems. However, the bottleneck of this technology lies in the complex separation and purification processes of exosomes, extremely low yields, difficulty in controlling drug loading efficiency, and high costs, which severely restrict their current clinical translation and large-scale application.

Therefore, each of these three delivery systems has its own strengths in terms of carrier properties, targeting mechanisms, maturity, and application challenges. Table 1 systematically compares their core characteristics.

## 6. Effect of Curcumin on the Proliferation of Mesenchymal Stem Cells

The effect of curcumin on mesenchymal stem cell (MSC) proliferation shows significant concentration-dependent and experimental model-dependent effects.

In in vitro experiments, low concentrations of curcumin (typically <10 μM) usually promote proliferation and inhibit apoptosis, while high concentrations may inhibit cell growth or induce cytotoxicity. For example, treatment with nano-curcumin—a nanoparticle formulation of curcumin—at low concentrations (0.3–0.7 μM) significantly increases the proliferation rate of bone marrow-derived MSCs (BM-MSCs) and maintains high cell viability by reducing apoptotic cell death [28]. Furthermore, the study found that low concentrations of curcumin can upregulate the expression of pluripotency genes such as OCT4 and SOX2, which are closely related to the self-renewal and proliferation of MSCs [28]. The bioconjugate formed by curcumin and epidermal growth factor (EGF), known as EGF-Cur B, was applied to MSC cultures, resulting in a significant enhancement of MSCs’ proliferative activity and the expression of pluripotency genes such as OCT4, SOX2, and Nanog, thereby promoting cell proliferation and maintaining stem cell characteristics [29]. However, high concentrations of curcumin (typically >20 μM) tend to cause cell cycle arrest, particularly at the G1 phase, and inhibit proliferation. These effects may stem from curcumin’s cytotoxicity or excessive induction of differentiation. This concentration threshold varies slightly among different cell types. For example, in human BM-MSCs, continuous exposure to 10 μM reduces proliferation, while 5 μM does not significantly affect proliferation but enhances differentiation potential [30]. In human umbilical cord Wharton’s jelly-derived MSCs (WJ-MSCs), curcumin maintains high cell viability in the range of 0.1–10 μM, while concentrations above 50 μM induce significant toxicity [31]. Curcumin exhibits a characteristic dose-dependent biphasic effect. The phenotypic differences observed in various studies essentially result from its transition between promoting proliferation at low concentrations and inhibiting growth at high concentrations. The concentration threshold for these effects is influenced by factors such as cell source and culture conditions. This reflects the complex biological activity of curcumin as a multi-targeted natural compound.

In animal experimental models, curcumin exhibits significantly low bioavailability due to its rapid metabolism and poor solubility after systemic administration [32]. As a result, it is difficult to achieve the precise and stable effective concentration required for in vitro cellular studies in target tissues. Consequently, related studies have observed that the core mechanism of action of curcumin in vivo is not to directly regulate the proliferation of target cells, but rather to create a more favorable microenvironment for the activation of endogenous MSCs or the transplantation of exogenous MSCs, through its powerful systemic anti-inflammatory, antioxidant, and anti-catabolic effects [33]. This regulation of the microenvironment, such as by modulating systemic and local inflammation and oxidative stress levels, indirectly supports the survival of MSCs and their repair functions, for example, promoting bone or cartilage repair.

In summary, the effect of curcumin on MSC proliferation exhibits a clear biphasic effect dependent on concentration; this has been well supported by evidence in in vitro experiments: low concentrations (typically <10 μM) generally promote proliferation and enhance pluripotent gene expression, while high concentrations (typically >20 μM) inhibit growth or induce toxicity, with specific thresholds varying based on cell source and culture conditions. This dose–response pattern directly reflects the multi-targeted biological activity of curcumin; however, its in vivo effects mainly stem from systemic effects, such as anti-inflammatory and antioxidant effects that improve the microenvironment, thereby indirectly supporting MSCs’ survival and function rather than directly regulating proliferation. While these studies provide valuable insights, uncertainties remain, including heterogeneity in the precise mechanisms (such as signaling pathways) among different MSC types and insufficient evidence of direct proliferation effects in vivo due to bioavailability limitations. Moreover, the regulatory balance between differentiation and toxicity at high concentrations remains partially speculative. Future research should focus on optimizing drug delivery systems to verify direct in vivo effects and elucidate the molecular basis behind cell-specific responses.

## 7. Curcumin Regulates the Apoptosis of Mesenchymal Stem Cells

MSCs have become a core cell resource in regenerative medicine due to their easy accessibility, low immunogenicity, and strong paracrine signaling functions. However, cellular senescence of MSCs that occurs during in vitro expansion significantly impairs their function. Additionally, after transplantation to damaged sites, MSCs encounter harsh microenvironments such as ischemia, hypoxia, and inflammation, which can lead to significant apoptosis in MSCs and severely limit their therapeutic efficacy. In this context, the regulatory effect of curcumin on MSC apoptosis has received increasing attention in recent years.

### 7.1. Curcumin Regulates the Apoptosis of BM-MSCs

BM-MSCs are the most widely studied type of MSCs. Research shows that curcumin can reduce the apoptosis of BM-MSCs by inhibiting oxidative stress and the inflammatory response. Yagi et al. found that the combined treatment of curcumin and Epigallocatechin gallate (EGCG) significantly inhibits H_2_O_2_-induced apoptosis of BM-MSCs and reduces the levels of ROS; this effect involves the protective role of the antioxidant enzyme system [34]. In addition, Sabouni et al. reported that nano-curcumin—a nanoparticle formulation of curcumin—can promote the proliferation of BM-MSCs and inhibit apoptosis at low concentrations (0.3–0.7 μM), while high concentrations induce apoptosis and inhibit proliferation [28]. Continuous exposure to 25 μM curcumin can inhibit the proliferation of BM-MSCs and increase the apoptosis rate, indicating that its effect is concentration-dependent [28]. Moreover, another study showed that curcumin pretreatment can reduce H_2_O_2_-induced apoptosis of BM-MSCs by inducing the expression of heme oxygenase-1 (HO-1) via the ERK1/2 signaling pathway [35].

### 7.2. Curcumin Regulates the Apoptosis of Adipose-Derived MSCs

Adipose-derived MSCs (AD-MSCs) have attracted attention due to their easy accessibility and strong proliferative capacity. Yousefi et al. found that low concentrations of nano-curcumin (0.3–0.7 μM) significantly inhibited the apoptosis of AD-MSCs, enhanced their proliferative capacity, and downregulated the expression of inflammatory factors, while high concentrations induced apoptosis, suggesting a concentration-dependent dual regulatory effect of curcumin [36]. Cremers et al. further confirmed that curcumin protects AD-MSCs from H_2_O_2_-induced apoptosis by activating the HO-1/CO pathway. This protective effect still exists in the HO-2 gene knockout model, indicating the core role of HO-1 [35].

### 7.3. Curcumin Regulates the Apoptosis of Umbilical Cord-Derived MSCs

Umbilical cord-derived MSCs (UC-MSCs) have low immunogenicity and high proliferation potential. Wu et al. reported that curcumin inhibits TNF-α-induced apoptosis of UC-MSCs in a dose-dependent manner, and this effect is mediated by the activation of the ERK1/2 signaling pathway. In vivo experiments further showed that the combined transplantation of curcumin and UC-MSCs can improve cell survival and enhance motor function after spinal cord injury [37]. Jinfeng et al. found that the conditioned medium of UC-MSCs pretreated with curcumin can more effectively reduce MPP+-induced apoptosis of PC12 cells and promote dopaminergic neuron differentiation compared to conditioned medium from untreated UC-MSCs; this effect is associated with increased secretion of anti-inflammatory and neurotrophic factors [38].

### 7.4. The Mechanism of Curcumin Regulating the Apoptosis of MSCs

The mechanism by which curcumin regulates MSC apoptosis involves multiple signaling pathways and molecular targets: ① antioxidant pathway: mitigating oxidative stress-induced injury by clearing ROS and upregulating antioxidant enzymes, including superoxide dismutase (SOD) [34]; ② anti-inflammatory pathway: inhibiting NF-κB activation and reducing the expression of proinflammatory cytokines such as IL-6 and TNF-α [39,40]; ③ mitochondrial pathway: regulating the Bcl-2/Bax ratio and inhibiting caspase-3 activation [30,41]; ④ epigenetic regulation: demethylation promotes the expression of miR-124 and miR-143, thereby inhibiting pro-apoptotic signaling pathways involving NF-κB and ROCK1 [42]. In addition, the nanoformulation of curcumin (such as nano-curcumin) can enhance its bioavailability and targeted delivery, strengthening its protective effect on MSCs [28,36].

In summary, curcumin regulates the apoptosis of MSCs from different sources through multiple pathways, and its effect is concentration-dependent: low concentrations (usually <10 μM) inhibit apoptosis, while high concentrations (usually >20 μM) induce apoptosis. However, the regulatory effects on MSCs from different sources are not homogeneous. These differences mainly stem from three factors: ① Tissue-specific microenvironment memory: MSCs from different sources retain characteristics imprinted by their native tissue microenvironment—for example, hypoxia in bone marrow, metabolic activity in fat, and fetal conditions in the umbilical cord. This leads to differences in their basic metabolic state, receptor expression, and sensitivity to signaling pathways. ② Selection of research models and stressors: Studies on MSCs from different sources often employ disease models most relevant to their clinical applications—such as BM-MSCs for bone repair, AD-MSCs for metabolic inflammation, and UC-MSCs for ischemic diseases. Additionally, the apoptosis inducers used (H_2_O_2_, TNF-α, serum deprivation) vary across studies, resulting in MSCs exhibiting different protective mechanisms in response. ③ The biological characteristics of the cells themselves: The proliferation capacity, aging rate, and secretion profile of MSCs from different sources differ, which may influence their metabolism and response threshold to curcumin.

There are still significant uncertainties and controversies regarding the effect of curcumin on the apoptosis of MSCs. Although the concept of low protection and high induction is widely accepted, there is no unified standard for the optimal protective concentration or the critical concentration that induces apoptosis for MSCs from specific sources. Moreover, the specific values reported in studies vary considerably. Additionally, most experimental data come from short-term in vitro treatment studies. Data on curcumin metabolites, interactions with host cells, and the long-term dynamic effects on MSC apoptosis remain very limited. Existing studies are mostly in vitro experiments, and in vivo applications require further exploration. Future research should focus on optimizing curcumin administration protocols—including dosage, delivery methods such as nanoparticle delivery, frequency, and formulation types—developing combined therapeutic strategies involving stem cell transplantation with curcumin, and conducting preclinical validation for specific disease models such as neurodegenerative diseases and liver injury. In addition, the synergistic effects of curcumin with other bioactive molecules, such as crocin, warrant further investigation.

## 8. Effect of Curcumin on Differentiation of Mesenchymal Stem Cells

### 8.1. Promotion of MSCs’ Osteogenic Differentiation

Curcumin can significantly promote the osteogenic differentiation of mesenchymal stem cells, and this effect offers valuable research directions and theoretical references for the fields of bone tissue engineering and regenerative medicine. Firstly, curcumin enhances the osteogenic differentiation of MSCs by directly regulating the expression of osteogenic-related genes. In rat bone marrow MSCs, curcumin treatment significantly increases alkaline phosphatase (ALP) activity and upregulates the gene expression of the key osteogenic transcription factor Runx2, as well as osteocalcin, thereby promoting the formation of mineralized nodules [43]. In human MSCs, curcumin alleviates the inhibition of osteogenic differentiation by downregulating the expression of the histone methyltransferase EZH2, which enhances the expression of osteogenic markers such as Runx2, Osterix, type I collagen, osteopontin, and osteocalcin [44]. In addition, curcumin induces early osteogenic differentiation of dental pulp stem cells (DPSCs), evidenced by the increased ALP activity and gene expression; this effect is more pronounced when combined with calcitriol [45].

Secondly, curcumin promotes osteogenic differentiation by regulating signaling pathways. Studies have shown that curcumin can activate the Wnt/β-catenin signaling pathway, This activation alleviates the inhibitory effect of oxidative stress on the osteogenic differentiation of the MSCs [46]. Moreover, curcumin regulates matrix metalloproteinases (MMPs). For example, it upregulates the expression of MMP-13 and inhibits MMP-1, thereby enhancing osteogenic differentiation and inhibiting adipogenesis in human BM-MSCs [30].

Curcumin not only directly acts on MSCs but also indirectly promotes osteogenic differentiation through immune regulation. Curcumin can polarize macrophages toward the M2 anti-inflammatory phenotype (M2 type) and increase the secretion of factors such as interleukin-4 (IL-4), IL-10, and bone morphogenetic protein-2 (BMP-2). Thus, in the co-culture system of macrophages and BM-MSCs, the osteogenic differentiation of BM-MSCs is significantly enhanced. This enhancement is evidenced by increased ALP activity and elevated expression of Runx2 and osteocalcin, which are markers of osteogenic differentiation [47].

**Figure 2:**
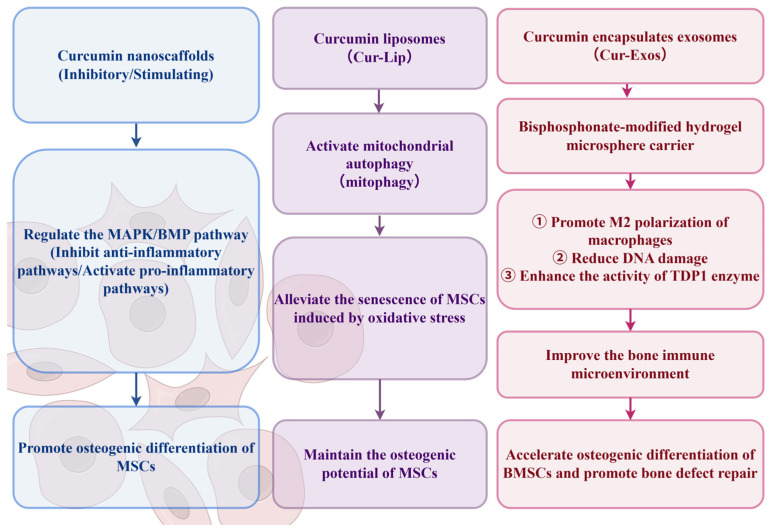
The mechanism of action of curcumin in regulating the osteogenic function of mesenchymal stem cells through different delivery systems (by figdraw.com).

Furthermore, the application of curcumin in nano-formulations enhances its bioavailability and efficacy, as shown in Figure 2. For example, curcumin-containing nanoscaffolds can promote the osteogenic differentiation of MSCs by modulating both inhibitory and stimulatory signaling pathways such as the MAPK and BMP pathways [48]. Curcumin liposomes (Cur-Lip) alleviate oxidative stress-induced senescence in MSCs by activating mitophagy, thereby maintaining their osteogenic potential [49]. Curcumin-encapsulated exosomes incorporated into bisphosphonate-modified hydrogel microspheres promote the M2 polarization of macrophages. Additionally, they improve the bone immune microenvironment by reducing DNA damage and enhancing TDP1 enzyme activity. These effects collectively accelerate the osteogenic differentiation of BM-MSCs and facilitate the repair of bone defects [50].

### 8.2. Promotion of MSCs’ Chondrogenic Differentiation

Curcumin can promote the differentiation of MSCs into chondrocytes by inhibiting the signaling pathways mediated by inflammatory factors. In the inflammatory environment stimulated by IL-1β, curcumin can inhibit the activation of NF-κB in a concentration-dependent and time-dependent manner; this reduces the expression of caspase-3 and cyclooxygenase-2 (COX-2) and increases the production of type II collagen, cartilage-specific proteoglycans (CSPGs), and β1-integrin, ultimately improving the chondrogenic differentiation potential of MSCs [39]. Moreover, curcumin also plays a key role in regulating the phenotypic stability of chondrocytes. Studies have shown that although curcumin does not directly affect the expression of chondrogenic markers (such as Sox9 and Col2a1), it can downregulate hypertrophic chondrocyte markers (such as Runx2 and Col10a1) by inhibiting the Indian hedgehog homolog (IHH) and Notch signaling pathways, thereby preventing MSCs from differentiating into hypertrophic chondrocytes and promoting the formation of a stable chondrocyte phenotype [51].

### 8.3. Inhibition of MSCs’ Adipogenic Differentiation

In BM-MSCs, continuous exposure to 5 μM curcumin can inhibit adipogenic differentiation while promoting osteogenic differentiation; this effect is related to the expression and activity of MMP-13 [30]. In the rat MSCs model, curcumin treatment not only reduces the mRNA expression of adipocyte-specific markers (such as PPARγ2 and C/EBPα) but also inhibits adipogenic differentiation by inducing the expression of HO-1, thereby promoting osteogenic differentiation [43]. Moreover, under oxidative stress conditions, curcumin can activate the Wnt/β-catenin signaling pathway, thereby reversing the inhibitory effect of oxidative stress on adipogenic differentiation [49].

## 9. Curcumin Regulates Factors Influencing MSCs

Curcumin shows significant dose-dependent biological effects on MSCs, and its effects are influenced by the source of MSCs, exposure time, drug delivery system, and microenvironmental factors.

### 9.1. Cell Sources

In BM-MSCs, curcumin enhances the osteogenic differentiation ability of BM-MSCs by regulating macrophage polarization and promoting the secretion of osteogenic-related factors such as BMP-2 and TGF-β; however, its effective dose must be strictly controlled to avoid cytotoxicity [47]. For adipose-derived MSCs (AD-MSCs), studies show that pre-treatment with 5 μM curcumin for 24 h can significantly inhibit the accumulation of ROS and cellular senescence, improving their antioxidant and anti-inflammatory capabilities through the FoxO3–autophagy signaling pathway. Higher doses, however, may induce cellular stress responses [52,53]. Regarding WJ-MSCs, curcumin intervention combined with these cells can regulate microglial polarization through the AKT/GSK-3β/β-TrCP/Nrf2 axis, thereby alleviating neuroinflammation after ischemia–reperfusion injury. Notably, the suitable dosage range for WJ-MSCs may differ from that of BM-MSCs and AD-MSCs due to the unique metabolic and signaling pathway characteristics of MSCs from different tissue sources [54].

### 9.2. Exposure Time

Exposure time is also a key factor affecting the effects of curcumin. Short-term treatment with curcumin usually enhances the migration, paracrine function, and/or stress resistance of MSCs, while long-term exposure may lead to autophagy activation and/or cell cycle arrest [52]. Therefore, a 24 h pre-treatment is commonly used, as it is sufficient for curcumin to regulate key signaling pathways within the cells [53,55].

### 9.3. Delivery System

The low bioavailability of curcumin limits its direct application, making the optimization of delivery systems particularly important. Studies have shown that nano-curcumin, liposomal curcumin, and exosomal curcumin can significantly improve the stability, targeting, and intracellular concentration of curcumin, thereby achieving effective therapeutic effects at lower doses. For example, exosomes loaded with curcumin derived from AD-MSCs, referred to as sEV-CUR, when delivered into the joint cavity, can alleviate oxidative stress and chondrocyte apoptosis in osteoarthritis [56]. Additionally, DNA tetrahedron-based nanocarriers can enhance osteogenic differentiation in BM-MSCs by targeting the ferroptosis pathway, which plays a role in regulating cell death and differentiation [57].

### 9.4. Microenvironment

Microenvironmental factors such as inflammation, oxidative stress, or high-glucose conditions can also regulate the effects of curcumin on MSCs. In diabetes or osteoporosis models, curcumin alleviates inflammatory responses by inhibiting the NF-κB and MAPK pathways, while promoting the differentiation of MSCs into osteogenic or neurogenic lineages [54,57]. It is noteworthy that MSCs from different sources respond heterogeneously to the microenvironment: AD-MSCs exhibit stronger angiogenic potential in skin wound healing [53], whereas BM-MSCs are more effective in bone repair [47,58]. This difference is closely related to their inherent transcriptomic profiles and secretory characteristics.

Therefore, the regulation of MSCs by curcumin is a process dependent on multiple factors. To maximize its therapeutic potential, the dose, timing, and delivery methods must be carefully designed and tailored to specific application scenarios, taking into account the heterogeneity of MSCs’ responses.

## 10. Research Limitations of Curcumin in Regulating MSCs

Although the regulatory effect of curcumin on MSCs has been demonstrated in many in vitro experiments and animal models, some problems still need to be addressed urgently.

First, low bioavailability is a major limiting factor for the preclinical studies of curcumin. Due to its poor water solubility and rapid metabolism by the liver, the in vivo half-life of curcumin is only about 1 to 2 h, resulting in significantly reduced bioavailability. The advancement of nanotechnology has offered novel approaches to address this issue; however, current nano-delivery systems still face many challenges in terms of stability, targeting, and large-scale production.

In addition, different nano-delivery systems have significant differences in their effects on MSC functions. These differences requires further research to clarify. Second, the dose dependence and the environmental specificity are important characteristics of curcumin in regulating MSCs. Generally, low-concentration curcumin can promote the proliferation and differentiation of MSCs, while high-concentration curcumin may inhibit cell functions and even induce apoptosis. At the same time, the regulatory effect of curcumin is environmentally specific and may produce different effects under different pathological conditions. This dose and environment dependence increases the complexity of clinical application and requires more in-depth research to establish the optimal treatment plan.

In addition, the complexity of the mechanism of action is another major challenge faced by curcumin in regulating MSCs. Curcumin regulates the functions of MSCs through multiple signal transduction pathways and various molecular targets, with complex interactions between these pathways and targets. Although this multi-target effect enhances the therapeutic potential of curcumin, it also increases the difficulty of mechanistic research. Consequently, it adds to uncertainty of clinical application.

Finally, the limitations of research models also affect the research progress of curcumin in regulating MSCs. Most current studies rely on in vitro cell culture and animal models, which differ in certain aspects from actual clinical situations. For example, the pathophysiological processes in animal models may not be completely the same as those in human diseases, which poses challenges for translating research results to clinical practice. In addition, MSCs from different species may have different responses to curcumin, which should be carefully considered in preclinical research.

Quality control and standardization are important issues that must be addressed in the clinical application of curcumin. The extraction and preparation processes of curcumin may affect its purity and activity; moreover, quality differences can exist between different batches. Additionally, variations in the concentration, treatment duration, and administration methods of curcumin used in different studies makes it difficult to compare and integrate the research results. Therefore, establishing unified quality control standards and standardized research methods is crucial for advancing the research and clinical application of curcumin in regulating MSCs.

## 11. Conclusions

As a substance with both nutritional and medicinal origins, curcumin demonstrates good biosafety. It can effectively promote the proliferation of MSCs, inhibit apoptosis, induce MSCs to differentiate into osteoblasts and chondrocytes, and inhibit their differentiation into adipocytes, thereby showing potential to enhance the therapeutic effects of MSCs. Its effects are influenced by multiple factors including cell source, exposure time, delivery systems, and microenvironment. However, current evidence mainly comes from in vitro experiments and animal studies, and curcumin itself faces known challenges such as low bioavailability and difficulties associated with effective in vivo delivery. Therefore, its clinical translation still needs to overcome many obstacles related to these challenges. To address these issues, future research could focus on dosage form optimization and improvement of delivery strategies; this would facilitate its transition from preclinical exploration to clinical application, providing new therapeutic options for the treatment of various diseases.

## Figures and Tables

**Table 1:** Differences between nano-curcumin, liposomal curcumin, and exosomal curcumin delivery systems.

Characteristics	Nano-Curcumin	Liposomal Curcumin	Exosomal Curcumin
Carrier nature	Artificially synthesized nanoparticles	Phospholipid biomimetic vesicles	Natural bio-derived nano-sized vesicles
Targeting mechanism	Passive (EPR effect)	Passive + modifiable to active targeting	Natural active targeting + engineerable modification
Biocompatibility	Moderate (depends on materials)	High	Extremely high
Large-scale production	Relatively simple and mature	Relatively mature, but stability is a key challenge	Extremely difficult, high cost, difficult to standardize
Main advantages	Simple process, significantly improves solubility	High encapsulation rate, good bio-membrane fusion, functionalizable	Low immunogenicity, excellent targeting and barrier penetration capability
Main limitations	Lack of active targeting, easily cleared by the body	Poor physicochemical stability, prone to leakage	Low yield, difficult separation and purification, challenging drug loading efficiency control
Technical maturity	High (already applied in food and health products)	Medium-high (already marketed drugs)	Low (mainly in preclinical research stage)

## Data Availability

No new data were created or analyzed in this study. Data sharing is not applicable to this article.
